# Case of Spina Bifida

**Published:** 1818-03

**Authors:** Pliny Hayes


*For the London Medical and Physical Journal.*

### *Case of Spina Bifida;* by Pliny Hayes, M.D.

On the 26th of March, Mrs. N. was delivered of a female child, with Spina Bifida. It moved the inferior limbs with facility, but appeared to possess no power of controling the evacuations. From the second to the fourteenth day, it was frequently convulsed. At the suggestion of a medical friend, the tumour was punctured on the eighteenth day, and its contents, which consisted of a limpid fluid, partially evacuated. The operation was repeated on the twentieth, and again on the twenty-fourth, when the internal surface of the sac, constituting the tumour, was pricked with the point of the needle, and, after the fluid was evacuated, a compress and bandage applied. The emaciation, which commenced at birth, had now become so great, that the parents objected to any further attempts to remedy the disease. On the thirty-fourth day, however, the tumour was again punctured, the internal surface of the sac irritated with the needle, the fluid evacuated, and a pad applied; but no adhesion or inflammation followed. The punctures, except the first, were made with a round pack-thread needle; half a line in diameter. They always healed, so as not to be visible on the third day. About this time, manifest signs of water in the head were perceived. The child lingered till the sixty-second day, and died.

The alvine evacuations had uniformly been green, more or less inclining to black, and frothy, and took place, as well as the urinary discharge, whenever the child cried, or was disturbed. It was generally very restless; but a grain or two of calomel, which was occasionally given, had always the effect of composing it for a few hours, and rendering the alvine discharges more natural. A pulsation was once distinctly noticed in the tumour.

*Dissection.*—In all the vertebræ of the sacrum, that portion which should form the posterior part of the vertebral canal, was entirely wanting. Below the lumbar vertebræ, the dura-matral sheath of the spinal marrow, was protected posteriorly by the common integuments only, with which it was firmly united. It formed here a sac, which contained the *cauda equina* and the fluid before-mentioned, and constituted the tumour. The spinal marrow entered the superior extremity of the sac entire, but was immediately bifurcated, and, after giving off considerable nerves through the inferior lumbar and superior sacral foramina, ramified over the whole internal surface of the sac, but principally on the lateral parietes, giving off filaments from numerous points which converged to pass through the holes of the sacrum into the pelvis. The sac terminated about half-way down the sacrum. The vertebral canal was not more than half filled by the spinal marrow. The vertebræ of the sacrum were cut through, to demonstrate that they contained no cavity. The fact, that the sphincters and contiguous muscular organs were paralized, while the inferior extremities were moved at will, may be readily explained by a reference to the different origins of the nerves, which supply the respective parts.

In the ventricles of the cerebrum were found four or five ounces of fluid, more than half of which consisted of thick pus. The contiguous medullary substance was discoloured, and of a weak and somewhat gelatinous texture. Situated above the lateral ventricles were found two preternatural cavities, one in each hemisphere, extending nearly the whole length of the middle and posterior lobes of the cerebrum. Their internal surfaces were sound, in some places exhibiting distinctly the fibrous structure of the medullary substance. They contained a very little fluid, without any pus. The cerebellum, and all the parts in the base of the skull, were sound. The subject was emaciated to the last degree. The ossification of the cranium was very imperfect. Many other singular appearances of less importance were noticed.—*New-England Journal.*

